# Practitioner views and experiences of deferred consent in paediatric and neonatal emergency care trials: the connect study

**DOI:** 10.1186/1745-6215-14-S1-P104

**Published:** 2013-11-29

**Authors:** Kerry Woolfall, Lucy Frith, Carrol Gamble, Bridget Young

**Affiliations:** 1 University of Liverpool, Liverpool, UK

## Background

Deferred consent refers to when a patient is entered into a trial without prior consent. In 2008 UK legislation was amended to enable the use of deferred consent for paediatric emergency care trials in recognition of the practical and ethical difficulties of obtaining consent in an emergency situation. However, ambiguity about how to make the process of deferred consent acceptable to parents, children and practitioners remains. In particular, little is known about practitioners’ views and experiences of seeking deferred consent in this setting.

## Methods

This paper presents empirical data from an online survey (45 practitioners) and qualitative focus group interviews (5 groups, 13 practitioners) to explore the views and experiences of UK clinical trial practitioners sampled to include both those who had hands-on experience of recruiting patients within a deferred consent framework and those who had no such experience.

## Results

Practitioners’ views on deferred consent differed depending upon whether or not they had experience of the consent method. Practitioners who had not previously used deferred consent were negative about it; they were particularly concerned about the impact that seeking deferred consent upon the parent-practitioner relationship. In contrast, practitioners who had experience of using deferred consent described how families had been receptive to this consent method, including how deferred consent had improved recruitment, parental decision-making capacity and parent-practitioner relationships in the emergency care setting.

## Conclusions

The ethical implications of these findings will be considered. Recommendations will be offered to inform future trial design, training, research and recruitment practice.

